# Household Hints and Recipes

**Published:** 1877-07

**Authors:** 


### Household Hints & Recipes.

TO CLEAN VARNISHED PAINT.—Boil a pound of bran in 1 gallon of water an hour, and wash the paint with the bran water.

FRENCH LIP SALVE.—Mix 16 ounces of lard, 2 ounces of white wax, ½ ounce each of nitre and alum in fine powder; alkanet to color.

CAMPHOR ICE.—Oil of sweet almonds, 2 ounces; spermaceti, 4 ounces; white wax, 2 ounces; camphor, ½ ounce; melt them over water bath; run into moulds.

TO CLEAN WHITE SPAR ORNAMENTS.—Brush with "Javelle Water," rinse in plain water, wipe and dry in the sun. This will also clean marble very well.

TO REMOVE FRECKLES.—Dissolve 3 grains of borax in 5 drachms each of rose water and orange flower water; a very good remedy is equal parts of pure glycerine and rose water, applied every night and allowed to dry.

WASH TO CLEANSE THE HAIR AND SCALP.—One teaspoonful powdered borax, 1 tablespoonful spirits of hartshorn, 1 quart soft water. Mix all together and apply to the head with a soft sponge, then rub the head well with a dry towel. Use once a week.

BARBERS' SHAMPOO MIXTURE:—Dissolve 1 oz. salts of tartar in 1 quart of soft water; sprinkle freely on the head and rub well till a lather is formed; wash off with clean water. Bay rum can then be used if desired.

GOLDEN BROWN HAIR DYE.—A solution of sulphate of copper (blue vitriol) followed by a solution ferrocyanide of potassium, gives an extremely rich, golden brown to light hair, when the process is expertly managed.

TO TAKE RUST OUT OF STEEL.—Place the article in a bowl containing kerosene oil, or wrap the steel up in a soft cloth well saturated with kerosene; let it remain 24 hours or longer; then scour the rusty spots with brickdust. If badly rusted, use salt wet with hot vinegar; after scouring, rinse every particle of brickdust or salt off with hot water; dry thoroughly; then polish off with a clean flannel cloth and a little sweet oil.

BLONDE OR FLAXEN HAIR DYE.—Mix in 10 ounces distilled water, 1 ounce acetate of iron, 1 ounce nitrate of silver, and 2 ounces nitrate of bismuth; moisten the hair with this mixture, and, after an hour, touch it with a mixture of equal parts of sulphide of potassium and distilled water.

BLEACHING LEAVES.—To bleach leaves, mix one drachm chloride of lime with one pint of water, and add sufficient acetic acid to liberate the chlorine. Steep the leaves about ten minutes, and until they are whitened; remove them on a piece of paper, and wash in clean water.

A NEW SOLVENT FOR COPAL.—Oleic acid is said to be an excellent solvent for gum-copal. On heating a small quantity of oleic acid it will dissolve a considerable quantity of copal. It is always a good means of distinguishing genuine amber from the imitation amber which always contains copal.

CHROMIC ACID FOR WARTS.—Three or four applications of this acid will cause the disappearance of warts, however hard, large, or dense these may be. The application gives rise to neither pain, suppuration, nor cicatrices, the sole inconvenience being the production of a dark brown color.

TO CLEAN SOILED RIBBONS AND SILKS.—A mixture of alcohol and highly rectified benzine is excellent for cleaning ribbons and silks. It is applied with a clean sponge. Persons must be careful not to use this mixture in an apartment where there is a fire or lamp burning.

VIOLET INK FOR RUBBER STAMPS:—

Aniline violet, 2 to 4 dr.
Alcohol, 15 dr.
Glycerine, 15 dr.

Dissolve. The solution is poured on the cushion and rubbed with a brush.

COCOANUT SOUP.—Grate six ounces of a perfectly sound cocoanut, put it into two quarts of strong veal stock, keep it closely covered and simmer one hour. Don't let it boil up strong; then strain through a fine sieve, so as to free from all the stringy substance of the cocoanut. Add fifty fine oysters, a gill of hot cream, a little flavoring of mace, or nutmeg, a very little cayenne pepper, salt to season to the taste; mix four even table spoonfuls of flour in a little cold water, make it perfectly smooth and free from lumps, and stir into the soup the last minute. Let it boil up once thoroughly and serve. This is a very palatable soup without the oysters.

TO PREVENT GLASS FROM BREAKING.—To avoid breakage of glass vessels when hot liquids or preserves are poured into them, set the vessel on a cloth previously dipped in cold water. This is singular, and to me inexplicably true. I have known and used the information for many years.—*Druggists' Advertiser.*

---

WRITING INK MARKS ARE REMOVED in various ways according to the sort of ink to be treated. Some are best obliterated by means of a solution of oxalic acid, others by chlorine water or an alkaline hypochlorite, and others again by nitro-muriatic acid. In all cases, the paper must afterwards be washed with several waters.

---

TO KEEP OIL CLOTHS LOOKING WELL.—Wash them once a month in skim milk and water—equal quantities of each. Rub them once in three months with boiled linseed oil. Put on very little, rub it well with a rag, and polish with a piece of old silk. Oil cloths will last years if kept in this way.

---

TO CLEAN PAINT.—Take 1 oz. pulverized borax, 1 lb. small pieces best brown soap, and 3 quarts water; let simmer till the soap is dissolved, stirring frequently. Do not let it boil. Use with a piece of old flannel, and rinse off as soon as the paint is clean. This mixture is also good for washing clothes.

---

EGG TEST.—The following is a simple but sure way to tell good from bad eggs:

Put them in water enough to cover them. All that lie flat, as they would on a smooth surface out of water, are good. Those of which the big end rises are bad. The vessel used should have a smooth, level bottom.

---

TO STEW CELERY.—Clean the heads thoroughly; take off the course green outer leaves; cut in small pieces, and stew in a little broth. When tender, add some rich cream, a little flour and butter, enough to thicken the cream. Season with pepper, salt, and a little nutmeg, if that is agreeable.

---

DRIED BEEF.—Slice the beef as thin as possible; put in a sauce pan, cover with cold water, and set over the fire till it slowly comes to a boil, then drain off all the water, add two gills of rich cream, if you have it, or rich milk, adding two tablespoonfuls of butter. If milk is used, wet to a smooth paste or cream a teaspoon and a half of flour and stir in as it comes to a boil, and serve hot.

TAR-WATER AS A DYE.—Tar-water may be employed for dyeing silk or wool ashen gray. The stuff is first mordanted with weak perchloride of iron, by soaking in the solution some hours. It is then drained and passed through the bath of tar-water. The oxyphenate of iron, which is thus precipitated on the fabric, gives a very solid color.

---

CEMENT FOR GLASS.—For mending valuable, glass objects, which would be disfigured by common cement, chrome cement may be used. This is a mixture of five parts gelatin to one of a solution of acid chromate of lime. The broken edges are covered with this, pressed together, and exposed to sunlight, the effect of the latter being to render the compound insoluble even in boiling water.

---

TO REMOVE STAINS FROM KID GLOVES.—Stains may be removed, even from the most delicately colored gloves, by suspending them for a day in an atmosphere of ammonia. Provide a tall glass cylinder, in the bottom of which place strong aqua ammonia. Be careful to remove from the sides of the jar any ammonia that may have spattered upon them. Suspend the gloves to the stopper in the jar. The gloves must not come in contact with the liquid.

---

ALCOHOL AND COLD.—At a meeting given to the Good Templars of the English arctic expedition, Mr. William Malley, of the Alert, in relating his experiences, said that among the few men who escaped scurvy, and did any sledging worthy of notice, were four teetotalers, who enjoyed perfect immunity from all sickness, establishing beyond the shadow of a doubt that the intense cold of the polar regions could be well endured without stimulants.

---

BUTTER AND ITS SUBSTITUTES.—A patent has been taken out in France for the manufacture of margarine, and it is allowed to be retailed on the condition that it is not described as butter. It is asserted that from one manufactory in Paris, employing 400 men, margarine to the amount of $400,000 a month is sold. It is principally used in France for kitchen purposes and by pastry cooks; but it is also bought in large quantities by the lower and poorer classes.

---

MOCK SWEETBREADS. — Three-quarters of a pound of veal well beaten in a marble mortar, a little suet, or bacon, the yolks of two eggs, and a spoonful (scant) of bread crumbs. Mix all well together, season with pepper, a very little mace and salt; add a great spoonful of cream. Roll up this compound in the shape of sweetbreads, and brown before the fire or in the oven. Serve with a gravy of brown flour, wet up with cream; stir into the gravy in the bake pan till smooth; then let it boil up once. If too thick add more cream, or a little boiling water.

---

"A RESEAT FORE THE SISTUM."—The following "Reseat" was given by a traveling doctor to a countryman, who paid him ten dollars for it and his advice:

Sasaprile thread of, 0 ʒ

Wahoon thread of, 0 ʒ

Wild Cherry quartr of, 0 ʒ

One pint of Alkhol.

2 pound of Shugr.

One pint of wattr.

Conmlit All.
pr dos one swaller 3 times pr dy Before Eating.

---

WASHING SUGGESTIONS.—The quickest and most thorough way of having a week's wash for six or eight people done: Do not boil the clothes; use two tablespoonfuls of powdered borax to every tubful of clothes; have the water hot; after one rubbing, let them soak for a short time in a tub of clean boiling water containing borax; use very little soap; rinse in one clear water. Washing can be done quicker and better if the clothes are put to soak overnight, in borax water, not soaped.—*Reader.*

---

INDIGESTIBLE SUBSTANCES.—The question: "How does dessiccation convert steamed grains into a wood-like substance?" propounded in the *Times*, by the manufacturers of steam cereals, is easily answered. The gluten, softened by steam, is "fixed" or permanently solidified by drying, and is forever afterward insoluble and indigestible. The same effect is produced upon animal albumen —a kindred substance—and upon gelatine. Modern chemistry teaches all this, and the experiment is easily tried. The richer the grain treated is in gluten, the more completely insoluble it is rendered. Thus wheat, steamed and dried, is nearly ruined in aroma, flavor, and assimilable nutriment, while oats are less seriously impaired, though rendered insipid and tasteless. The theory that the cereal grains can be in any way improved or their nutritive elements added to by steaming and subsequent drying, is something more serious than fallicy, since it may induce ignorant persons to employ them as foods, and to suffer by their use.—*Physician, N. Y. Times.*

PRESERVATION OF FOOD.—Carbolic acid paper, which is now much used for packing fresh meats for the purpose of preserving them against spoiling, is made by melting five parts of stearine at a gentle heat and then stirring in thoroughly two parts of carbolic acid; after which five parts of melted paraffine are to be added.

The whole is to be well stirred together until it cools, after which it is melted and applied with a brush to the paper in quires, in the same way as in preparing the waxed paper so much used in Europe for wrapping various articles.—*Journ. Soc. Arts.*

---

TO PURIFY A SICK CHAMBER.—The nitrous acid vapor, so invaluable as a disinfectant in contagious fevers, is obtained by decomposing nitre by means of heated sulphuric acid, in the following manner:

Put ½ ounce sulphuric acid in a crucible glass or china cup and warm it over a lamp or in heated sand, adding to it from time to time a little nitre. Place several of these vessels in the sick chamber and in the neighboring apartments and passages, at a distance of 20 feet or more from each other, according to the height of the ceiling and the virulence of the contagion.

---

PRESERVING EGGS.—Among recent experiments made in Bavaria, relating to the preservation of animal food, the military authorities have derived great satisfaction from those in the desiccation of eggs. These can be preserved in a dried state, retaining all their nutritive qualities for transportation. The best process of preserving eggs which has yet come to our notice, is the following, which we glean from the *English Mechanic;* the writer says:—In the year 1871-72, I preserved eggs so perfectly that, after a lapse of six months, they were mistaken when brought to table for fresh-laid eggs, and I believe they would have kept equally good for twelve months. My mode of preservation was to varnish the eggs as soon after they were laid as possible with a thin copal varnish, taking care that the whole of the shell was covered with the varnish. I subsequently found that by painting the eggs with fresh albumen, beaten up with a little salt, they were preserved well, and for as long a period. After varnishing or painting with albumen, I lay the eggs upon rough blotting paper, as I found that when allowed to rest till dry upon a plate or on the table, the albumen stuck so fast to the table or plate as to take away a chip out of the shell. This is entirely obviated by the use of the blotting paper. I pack the eggs in boxes of dry bran.—*Druggists' Advertiser.*

TO DRY APPLES, PEARS AND OTHER FRUITS.—Have a frame made in the following manner:

Two strips of board 7 feet long, 2 or 2½ inches wide; two strips 3 feet long, 1½ inches wide, the whole ¾ of an inch thick; nail the long strips across the ends of the short ones, and it makes a frame 7 by 3 feet, which is a convenient size for all purposes. On one of the long strips, nails are driven 3 inches apart, extending from one end to the other. After the apples are pared they are quartered and cored, and with a needle and twine, or stout thread, strung into lengths long enough to reach twice across the frame; the ends of the twine are then tied together, and the strings hung on the nails across the frame. The apples will soon dry so that the strings can be doubled on the nails, and fresh ones put on, or the whole of them removed and others put in their place. As fast as the apples become sufficiently dry they can be taken from the strings, and the same strings used to dry more on. Pears and other fruit can be dried in this way.

---

WHITEWASHING.—According to the *English Mechanic*, the following is a correct scientific and practical rule: Well wash the ceiling by wetting it twice with water, laying on as much as can well be floated on, then rub the old color up with a stumpy brush and wipe off with a large sponge. When this is done, stop all the cracks with whiting and plaster of Paris. When dry, claircole with size and a little of the whitewash. If very much stained, when this is dry, paint those parts with turps, color, and, if necessary, claircole again.—To make the witewash, take a dozen pounds of whiting (in large balls), break them up in a pail, and cover with water to soak. During this time melt over a slow fire 4 lbs. common size, and at the same time, with a palette knife or small trowel, rub up fine about a desert-spoonful of blue-black with water to a fine paste; then pour the water off the top of the whiting, and with a stick stir in the black; when well mixed, stir in the melted size and strain. When cold it is fit for use. If the jelly is too stiff for use, beat it well up and add a little cold water. Commence whitewashing over the window, and so work from the light; lay off the work into that done, and not all in one direction, as in painting. Distemper color of any tint may be made by using any other color instead of the blue-black—as ochre, chrome, Dutch pink, raw sienna for yellows, red, buff; venetian red, burnt sienna, Indian red, or purple brown for reds; celestial blue, ultramarine, indigo for blues; red and black for purple, gray, or lavender; red lead or chrome for orange; Brunswick green for greens.

WHITEWASH FOR OUT-DOOR USE.—The following is a very excellent recipe, and is said to be used by the Government (in non-political jobs):

Lime, ½ bushel.
Salt, 1 peck.
Rice (ground) 3 pounds.
Spanish white ½ pound.
Glue 1 pound.
Water, sufficient quantity.

Slack the lime in a convenient vessel with *boiling* water, keeping the vessel covered during the operation. Strain the liquor through a fine sieve, and add the salt, previously dissolved in warm water, and the rice previously boiled to the consistency of a thin paste, add the whiting and the glue, which should be dissolved by soaking it in warm water. Place the mixture in a kettle, suspended within a larger one filled with water, on a slow fire; add five gallons of hot water to the mixture, and let it stand for a few days, covered to protect it from dust. It should be put on hot. About a pint of the mixture will cover a square yard of board surface if properly applied. This wash is said to answer quite as well as oil paint for wood, brick or stone, and it is much cheaper. It will retain its brilliancy for many years. Coloring matter may be added for any shade except green, for which there is no coloring material that can be used with lime. Spanish brown yields a reddish-pink shade when stirred in, more or less deep, according to the quantity used. Yellow ochre yields a yellow wash, but chrome goes further and makes a color generally better liked. Finely pulverized common clay, mixed with Spanish brown, makes a reddish stone color. Experiments in coloring had better be tried with small quantities, and allowed to dry after applying.—*Druggists' Advertiser*.

---

SOLVENT FOR RUBBER.—This new solvent consists of a mixture of methylated ether and petroleum spirit—the common benzolene used for burning in sponge lamps. This forms the most rapid and, perhaps, the best solvent we have tried; the mixture is as much superior in power to either of its constitutents singly as the ether-alcohol is to plain either in its action on paroxylin. We make a very thick solution by dissolving sixty grains of good india rubber in two ounces of benzolene and one ounce of sulphuric ether. If the india rubber be cut up fine and the mixture shaken occasionally, the solution will be complete in two or three hours, when it may be diluted to any required strength with benzolene alone. The india rubber should be as light colored as possible, and all the outer oxidized portions must be cut away. Shred the clean india rubber with a pair of scissors, and throw it at once into the solvent.—*British Journal of Photography*.

